# Case Series Evaluating the Use of Combined Long Acting Injectable Antipsychotics in Three Patients Within Forensic Services

**DOI:** 10.1192/bjo.2022.98

**Published:** 2022-06-20

**Authors:** Megan Moxon-Holt, Chris Todd

**Affiliations:** Devon Partnership NHS Trust, Exeter, United Kingdom

## Abstract

**Aims:**

Antipsychotic polypharmacy is a relatively common practice, despite a lack of robust evidence. In 2018, the National Clinical Audit of Psychosis evaluated the treatment of 8000 patients with a diagnosis of schizophrenia or schizoaffective disorder, and found 10% were receiving non-clozapine antipsychotic polypharmacy. This included 432 patients receiving one oral & one long acting injectable (LAI) antipsychotic and 2 patients receiving two LAI antipsychotics. An audit within our service, found that 24 of 88 (27%) inpatients were receiving non-clozapine antipsychotic polypharmacy. Of these, 3 were prescribed two LAI antipsychotics. A literature review found very limited evidence supporting the use of combined LAI antipsychotics, with publications relating to a total of 18 cases. Presented here is a case series, reviewing the use of LAI antipsychotic polypharmacy in three patients within Devon Partnership Trust.

**Methods:**

The case series reports on three male inpatients, who are under the care of secure services within Devon Partnership Trust. All are currently prescribed two LAI antipsychotics. Two have a diagnosis of treatment resistant schizophrenia and one of schizoaffective disorder. All are complex, necessitating recurrent or lengthy admissions, and present with significant risk to others when unwell. In each case, there have been trials of multiple antipsychotics, but only one has had a previous trial of clozapine.

**Results:**

Published case reports highlight the positive effects of LAI polypharmacy, noting an improvement in mental state and lack of adverse effects. The cases presented here show significant variability, with one patient improving significantly, the second to a lesser extent, and the third remaining under high level observations.

All cases are complex with decisions taken on a background of high risk, after multiple failed trials of medication.

Although no specific adverse effects were reported, none of the patients regained sufficient insight to engage in treatment decisions and physical health monitoring. It is therefore difficult to quantify the adverse effect burden and weigh this against perceived efficacy.

**Conclusion:**

Combined LAI antipsychotic medication is a possible treatment option in complex individual cases. Prescribing decisions are based on perceived clinical benefit, and the evidence base remains limited, with little understanding of long-term effects or consequences.

Unlike high dose antipsychotics, there is no formalised guidance for prescribing combined LAI antipsychotics. Treatment targets and review processes were not always explicit. A more robust approach, would provide greater clarity around the practice and aid with future decision making.

